# Decreasing trends in potentially inappropriate medications in older people: a nationwide repeated cross-sectional study

**DOI:** 10.1186/s12877-021-02568-1

**Published:** 2021-11-02

**Authors:** Solène Drusch, Thien Le Tri, Joël Ankri, Mahmoud Zureik, Marie Herr

**Affiliations:** 1 EPI-PHARE, epidemiology of health products (French National Agency for the Safety of Medicines and Health Products, and French National Health Insurance), 143 Boulevard Anatole, 93200 Saint-Denis, Paris, France; 2 University Paris-Saclay, UVSQ, Inserm, CESP, Anti-infective Evasion and Pharmacoepidemiology, 78180 Montigny le Bretonneux, France; 3 Epidemiology and Public Health department, AP-HP. University Paris-Saclay, Paris, France

**Keywords:** Potentially inappropriate medications, Polypharmacy, Aged people, Trends, Cross-sectional study

## Abstract

**Background:**

Potentially Inappropriate Medications (PIMs) and polypharmacy are widely used indicators of suboptimal prescribing for older people. The aim of this study was to describe the changes in the prevalence of PIMs and polypharmacy among people aged 75 years and over between 2011 and 2019 in France.

**Methods:**

PIMs and polypharmacy were assessed among people aged 75 years and over every two years between 2011 and 2019 using the French health insurance data system. Sixteen PIM criteria from the 2015 Beers and STOPP lists were assessed. Polypharmacy (5 to 9 drugs) and hyper-polypharmacy (≥10 drugs) were defined based on the average number of drugs dispensed per quarter. The Annual Percent Change (APC) and 95%CI were assessed using linear regression models after standardization of the prevalence on age and sex.

**Results:**

The study population included 5,777,645 individuals over 75 years old in 2011 and 6,328,155 in 2019. The prevalence of PIMs decreased from 49.6 to 39.6% over the study period (APC: − 1.19% [− 1.35;-1.04]). Of the sixteen indicators assessed, the prevalence of thirteen decreased between 2011 and 2019. Benzodiazepines were the most frequent PIMs (34.7% in 2011 to 26.9% in 2019), followed by anticholinergic drugs (12.1% in 2011 to 8.3% in 2019), oral non-steroidal anti-inflammatory drugs (11.4 to 7.8%), and PIMs related to antihypertensive drugs (7.4 to 6.0%). Overall, women and individuals aged 85 years and older were more likely to receive PIMs. The prevalence of hyper-polypharmacy decreased from 30.5 to 25.9% over the study period.

**Conclusion:**

This study, which is the first to assess the change in prevalence of PIMs and polypharmacy over time from comprehensive health data in France, highlights that PIMs and hyper-polypharmacy declined between 2011 and 2019. However, PIMs remains frequent for older people and often involves benzodiazepines.

**Supplementary Information:**

The online version contains supplementary material available at 10.1186/s12877-021-02568-1.

## Background

In the European Union, people aged 65 years and over are expected to account for 29% of the population by 2050, compared to 20% in 2019 [1]. Population aging is a global phenomenon which goes hand-in-hand with increasing morbidity and use of healthcare resources, including medications [2]. The concomitant use of multiple drugs is commonly referred to as polypharmacy, although thresholds to define polypharmacy vary [3]. In 2019, a review of literature indicated that the prevalence of polypharmacy ranged from 4 to 57% in older people [4]. Although medications can improve the health and life expectancy of older people, polypharmacy has been associated with increased risk of drug interactions, adverse health outcomes (e.g. falls, renal failure and frailty), hospitalization and medical costs [5]. However, polypharmacy may not be avoidable in a context of multimorbidity [6], and one way to prevent the occurrence of adverse events in older people may then be to focus on Potentially Inappropriate Medications (PIMs).

PIMs are defined as drugs with a poor benefit/risk ratio in older persons, mainly because of changes in the pharmacokinetics and pharmacodynamics properties of drugs with age [7]. Multiple PIM lists have been developed by expert panels to facilitate the identification of drugs, drug-drug or drug-disease interactions that should be avoided in older people. The Beers’ criteria were the first of these to be published, in 1991. Initially designed to be used in US nursing homes, their scope has been extended to older people living in community and they have been regularly updated since then by the American Geriatrics Society to include new recommendations [8]. Another popular PIM list was developed in Europe, namely the Screening Tool of Older People’s Prescriptions (STOPP) criteria [9]. Such tools can be used in practice but also in research to study the prevalence, risk factors and consequences of suboptimal prescribing. A meta-analysis of observational studies published between 2002 and 2019 estimated the pooled prevalence of PIMs to be 33.3% (95%CI: [29.7,37.0]) in people aged 65 or older in primary care, although there are some differences in the PIM lists used between studies [10]. This meta-analysis also estimated that PIMs may explain 15% of the risk of functional decline, 10% of the risk of adverse events, and 8% of the risk of hospitalization.

If we look at the evolution of the prevalence of PIMs and polypharmacy in recent years, conflicting results can be observed. On the one hand, several studies show that the prevalence of polypharmacy increased in Italy, Ireland, Sweden, New Zealand and the USA between 1990 and 2015 [11]. Considering that polypharmacy has been shown to be associated with an increased risk of inappropriate prescribing [12–14], one would also have expected an increase in PIM prevalence. However, to the best of our knowledge, no study has reported an increase in PIM prevalence over the past two decades. On the contrary, studies in the UK and the Netherlands show stable levels [12, 15] and studies in Europe and in the USA even report a decrease in the prevalence of PIMs [12–14, 16, 17]. In France, a study conducted between 1995 and 2004 indicated a downward trend [18]. More recent studies based on the French national insurance healthcare system estimated the PIM prevalence in people aged 75 years and over to be 53% in 2011 [19] and 32% in 2014 [20], but differences in population and methodology between studies prevent direct comparison.

Considering together the trends in the prevalence of PIMs and polypharmacy reported in the literature and the lack of recent evaluation in France, we sought to assess the change in the prevalence of PIMs and polypharmacy among older people in France between 2011 and 2019.

## Methods

### Study design

Using data from the French National Health Data System, we conducted a repeated cross-sectional study to assess the prevalence of PIMs and polypharmacy among older people every two calendar years between 2011 and 2019.

### Data sources

Data from the French health insurance claims data system (*Données de Consommation Inter-Régime* [DCIR]) were used. The French health insurance system is universal and registration is compulsory. Therefore, the DCIR covers about 99% of the French population (approximately 67 million people in 2019) from birth (or immigration) to death (or emigration). Each beneficiary is anonymously identified by a unique number. The database has been previously described and widely used for epidemiological studies [21, 22]. It contains information on socio-demographic characteristics (age, sex, place of residence, date of death), outpatient reimbursed medical care and reimbursed drugs dispensed in pharmacies. The drugs are coded according to the Anatomical Therapeutic Chemical (ATC) classification.

### Study population

The study population included people aged 75 years and over who had received at least one reimbursement for outpatient care in a given year (2011, 2013, 2015, 2017 and 2019). The same methodology was used for the selection of the study population each year to allow comparisons between years. We restricted the study population to people aged 75 years and over because of the increasing risk of adverse events with age and in continuity with previous work in France [19]. The study population included all people who met the inclusion criteria regardless of their living place (community or nursing home), as this information was not available in the DCIR. People who died during the study year were excluded in order to have a comparable length of observation for all individuals. Other reasons for exclusion were abnormal date of birth (prior to 1900), unknown sex, and temporary identifier (e.g. related to a recent immigration). The different steps of the selection of the study population are detailed in the online Supplementary Table 1.

### PIM criteria

PIM criteria involving drugs marketed and reimbursed in France were defined based on the 2015 updates of the Beers’ criteria [8], and STOPP criteria [9]. Because the DCIR only provides information on the reimbursement of ambulatory care, we excluded the criteria that could not be fully assessed, namely those based on drug-disease or drug-syndrome interactions, as well as other criteria requiring information about drugs’ indication or dosage (e.g. proton-pump inhibitors beyond 8 weeks without justification, or daily doses of digoxin > 0·125 mg). Our final set of criteria included sixteen criteria in four therapeutic areas: Benzodiazepines (BZDs), oral Non-Steroidal Anti-Inflammatory Drugs (NSAIDs), drugs with AntiCHolinergic effects (ACH) and AntiHyperTensive drugs (AHT). The different criteria and their rationale are presented in Table 1, additional information about the molecules and their ATC codes are available in the online Supplementary Table 2.

**Table 1:** Definition of PIM criteria included in the study, according to 2015 Beers and STOPP criteria

Indicator of potentially inappropriate medication	Source	Rationale
**Anticholinergic drugs**		Increased risk of: - Cognitive impairment, confusion, sedation - Orthostatic hypotension, falls - Urinary retention - Dry mouth - Constipation
Tricyclic antidepressants	Beers 2015 STOPP 2015	
Phenothiazines	Beers 2015 STOPP 2015	
First-generation antihistamines	Beers 2015	
**Non-steroidal anti-inflammatory drugs (NSAIDs)**		Increased risk of gastrointestinal bleeding or peptic ulcer disease. Increased risk of renal failure.
Three or more dispensations of oral NSAIDs	Beers 2015 STOPP 2015	
Concurrent use of two NSAIDs or more	STOPP 2015	No enhancement of efficacy
NSAIDs in combination with vitamin K antagonist or non-vitamin k antagonist oral anti-coagulant	STOPP 2015	
NSAIDs in combination with antiplatelet agents	STOPP 2015	
**Benzodiazepines**		For all BZDs, increased risk of: - Cognitive impairment - Delirium - Falls and fractures - Road accident
Other Benzodiazepines (Short- or intermediate- acting)	Beers 2015	
Long-acting benzodiazepines Three or more dispensations of other benzodiazepines (short-or intermediate-acting) (half-life ≥20 h)	Beers 2015	Decreased metabolism of long-acting benzodiazepines in old people, increased risk of adverse effects
Hypnotic Z-drugs	Beers 2015 STOPP 2015	Increased risk of hospitalization, minimal improvement in sleep latency and duration
Concurrent use of 2 or more benzodiazepines	Beers 2015 STOPP 2015	No improvement of efficacy and increased risk of adverse effects
Concurrent use of benzodiazepines and hypnotic Z-drugs	Beers 2015	Increased risk of adverse effects
Concurrent use of opioid receptor agonists and benzodiazepines	Beers 2015	Increased risk of fall and overdose
**Antihypertensive drugs**		
Central alpha-blockers	Beers 2015	Increased risk of : - Adverse central nervous system effects - Orthostatic hypotension - Bradycardia
Selective calcium channel blockers with immediate release	Beers 2015	Risk of hypotension and precipitating myocardial ischemia
Concurrent use of β-blockers and verapamil or diltiazem	STOPP 2015	Increased risk of heart block

PIMs were defined by at least one dispensation of the corresponding ATC code during the year, except for NSAIDs and short- and intermediate-acting BZDs for which potentially inappropriate use was defined from three dispensations during the year (not necessarily consecutive). Criteria related to drug-drug interactions were assessed by considering that drugs delivered on the same day are used concomitantly. This short time window is meant to prevent classification errors where two drugs are considered concomitant when one is actually used as a relay or replacement for another.

### Polypharmacy

Polypharmacy was defined as the annual average of the number of drugs with different ATC5 codes that were dispensed in a quarter [23]. Vaccines, homeopathy, diagnostics agents, antiseptics and disinfectants, medical gases, medicated and surgical dressings, blood substitutes and perfusion solutions, immune sera and immunoglobulins were excluded from the count of drugs. Polypharmacy was categorized into three classes: no polypharmacy (0 to 4 drugs), polypharmacy (5 to 9 drugs) and hyper-polypharmacy (≥ 10 drugs).

In addition, we defined an indicator of polypharmacy focusing on psychotropic medications according to the 2019 update of the Beers’ criteria [24]. This indicator is based on the concomitant dispensation (on the same day) of any combination of three or more Central Nervous System (CNS)-active drugs including antipsychotics, BZDs, hypnotic Z-drugs, tricyclic antidepressants, selective serotonin reuptake inhibitors, opioids and antiepileptic drugs.

### Analyses

The descriptive analyses report the prevalence of all individual PIM criteria and of polypharmacy in numbers and percentages. A composite indicator was also created to assess the overall exposure to PIMs, encompassing the 16 individual PIM criteria as well as psychotropic polypharmacy. In order to take into account demographic changes over the study period in the analysis, the prevalence of PIMs and polypharmacy were standardized based on the structure of the population in 2019. Furthermore, we used the sex-standardized prevalence of PIMs and polypharmacy to describe trends in old people (75 to 84 years) and in the oldest-old (85+) and the age-standardized prevalence to describe trends in men and women separately.

Changes in the prevalence of PIMs and polypharmacy during the study period were assessed using linear regression models with the logarithm of the prevalence as dependent variable and the study year as continuous explanatory variable. Under the assumption of linearity on the log scale, the parameter estimated by the linear regression (β) was transformed as follows, (exp(β)-1) × 100, to provide an estimation of the Annual Percent Change (APC) and its 95% Confidence Interval (95%CI) [25]. The APC estimates the relative change in the prevalence in a given year compared to the previous one. It is a relative measure that allows the comparison of changes between PIMs that do not have the same prevalence initially. In order to compare the APC between sex and age categories, we estimated the 95%CI of the difference in APC between men and women in one hand, and between the old and the oldest-old in the other hand.

Analyses were performed using SAS, version 9.4 software (SAS Institute, Inc) and RStudio for graphics (version 9.04).

## Results

### Study population

The size of the study population was 5,777,645 in 2011 and it reached 6,328,155 in 2019 (Table 2). The proportion of individuals with at least one drug dispensation over the year increased from 94.0% in 2011 to 96.5% in 2019. The proportion of oldest-old (aged 85 years and over) among the study population increased from 30.8% in 2011 to 35.9% in 2019. The sex ratio was stable, with 61% of women on average each year.

**Table 2:** Characteristics of people aged 75 years or over with a health care reimbursement, France, 2011–2019

		2011		2013		2015		2017		2019	
		(5,777,645)		(6,068,742)		(6,151,861)		(6,206,973)		(6,328,155)	
		N or mean	% or s-d	N or mean	% or s-d	N or mean	% or s-d	N or mean	% or s-d	N or mean	% or s-d
**Sex**											
	Men	2,181,209	37.8	2,338,065	38.5	2,395,745	38.9	2,448,459	39.5	2,535,597	40.1
	Women	3,596,436	62.3	3,730,677	61.5	3,756,116	61.1	3,758,514	60.6	3,792,558	59.9
**Age (years)**		82.1	5.4	82.4	5.5	82.6	5.6	82.8	5.7	82.8	5.8
	75 to 79	2,194,336	38.0	2,218,580	36.6	2,179,628	35.4	2,131,076	34.3	2,194,379	34.7
	80–84	1,806,207	31.3	1,887,832	31.1	1,887,550	30.7	1,872,629	30.2	1,861,395	29.4
	85–89	1,178,665	20.4	1,244,614	20.5	1,290,619	21.0	1,343,573	21.7	1,368,345	21.6
	Over 90	598,437	10.4	717,716	11.8	794,064	12.9	859,695	13.9	904,036	14.3
**At least one drug reimbursement**		5,430,172	94.0	5,764,924	95.0	5,900,245	95.9	5,948,385	95.8	6,106,212	96.5

### Changes in PIM prevalence

**Fig. 1:**
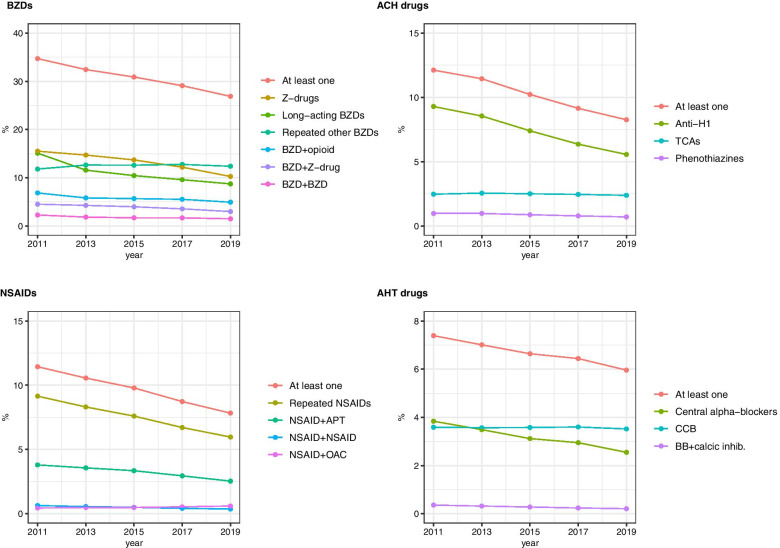
Trends in age- and sex-standardized prevalence of exposure to potentially inappropriate prescriptions of Nonsteroidal Anti-Inflammatory Drugs (NSAIDs), Benzodiazepines (BZDs), Anticholinergics drugs (ACH) and AntiHyperTensive drugs (AHT), France, 2011–2019. *Hypnotic Z-drugs (Z-drugs); Long-acting benzodiazepines (Long-acting BZDs); Short- and intermediate- acting benzodiazepines three or more dispensations during the year (Repeated other BZDs); Concurrent use of benzodiazepine and opioids (BZD + opioid); Concurrent use of at least two benzodiazepines (BZD + BZD); Concurrent use of benzodiazepines and hypnotic Z-drugs (BZD + Z-drug), First-generation antihistamine (ANTI-H1), Tricyclic antidepressants (TCAs); Concurrent use of two NSAIDs (NSAID + NSAID); NSAIDs three or more dispensations during the year (Repeated NSAIDs); Concurrent use of NSAID and antiplatelet agents (NSAID + APT); concurrent use of NSAID and vitamin K antagonists or non-vitamin k antagonist oral anti-coagulant (NSAID + OAC); concurrent use of β-blockers and verapamil or diltiazem (BB + calcic.inhib), selective calcium channel blockers with immediate release (CCB)*

The changes in PIM prevalence (overall and by category) are presented in Table 3 and Fig. 1. The overall prevalence of PIMs decreased from 49.6% in 2011 to 39.6% in 2019, corresponding to an overall relative decrease of 20% and an APC of − 1.19% per year (95%CI: [− 1.35 to − 1.04]). The prevalence of thirteen out of sixteen PIM criteria decreased significantly between 2011 and 2019, as did the crude number of users despite the increase in the population size over the study period.

**Table 3:** Age and sex-standardized prevalence of Potentially Inappropriate Medications (PIMs) dispensations by year and Annual Percent Change (APC), France, 2011–2019

		2011		2013		2015		2017		2019		APC^a^	95%CI	p-value for trend
		(5,777,645)		(6,068,742)		(6,151,861)		(6,206,973)		(6,328,155)				
		N	%	N	%	N	%	N	%	N	%	%		
**At least one PIM during the year**	Overall	2,884,533	49.6	2,872,892	47.2	2,776,084	45.0	2,639,705	42.4	2,506,266	39.6	−1.19	−1.35 to − 1.04	< 10^−3^
	75 to 84 years^b^	1,981,813	49.1	1,912,282	46.3	1,792,775	43.9	1,656,516	41.3	1,548,856	38.2	−1.33^d^	− 1.49 to − 1.18	< 10^− 3^
	≥85 years	902,720	50.5	960,610	48.7	983,309	47.0	983,189	44.5	957,410	42.1	−0.98	− 1.15 to − 0.80	< 10^− 3^
	Women^c^	1,979,027	54.9	1,953,753	52.3	1,885,850	50.2	1,787,788	47.6	168,9605	44.6	−1.11^d^	− 1.37 to −0.95	< 10^−3^
	Men	905,506	41.6	919,139	39.4	890,234	37.2	851,917	34.8	816,661	32.2	−1.37	−1.53 to − 1.20	< 10^−3^
**PIMs related to benzodiazepines**	Overall	2,018,057	34.7	1,972,800	32.4	1,904,170	30.9	1,810,059	29.1	1,700,774	26.9	−1.34	−1.50 to − 1.17	< 10^−3^
	75 to 84 years	1,350,688	33.3	1,265,982	30.6	1,178,821	28.8	1,083,488	27.0	997,878	24.6	−1.58^d^	−1.77 to − 1.38	< 10^−3^
	≥85 years	667,369	37.2	706,818	35.8	725,349	34.6	726,571	32.9	702,896	30.9	−0.98	−1.18 to − 0.79	< 10^−3^
	Women	1,450,852	40.4	1,407,923	37.9	1,355,991	36.2	1,284,959	34.2	1,205,028	31.8	−1.25^d^	−1.41 to − 1.10	< 10^−3^
	Men	567,205	26.2	564,877	24.3	548,179	23.0	525,100	21.5	495,746	19.6	−1.54	−1.74 to − 1.33	< 10^−3^
**PIMs related to anticholinergic drugs**	Overall	709,468	12.1	701,028	11.4	633,505	10.2	569,508	9.2	522,718	8.3	−2.13	−2.47 to − 1.78	< 10^− 3^
	75 to 84 years	499,332	12.4	485,706	11.7	429,227	10.5	380,713	9.5	349,344	8.6	−2.01^d^	−2.35 to −1.66	< 10^−3^
	≥85 years	210,136	11.7	215,322	10.9	204,278	9.8	188,795	8.6	173,374	7.6	−2.38	− 2.73 to − 2.02	< 10^− 3^
	Women	493,035	13.6	480,512	12.8	434,565	11.5	389,517	10.4	354,392	9.3	−2.07	− 2.37 to −1.76	< 10^−3^
	Men	216,433	9.9	220,516	9.4	198,940	8.3	179,991	7.3	168,326	6.6	−2.26	−2.69 to − 1.82	< 10^−3^
**PIMs related to non-steroidal anti-inflammatory drugs**	Overall	681,835	11.4	654,359	10.6	609,660	9.8	543,332	8.7	495,720	7.8	−2.04	−2.33 to −1.74	< 10^−3^
	75 to 84 years	524,764	13.0	498,100	12.1	460,543	11.3	406,396	10.1	369,103	9.1	−1.92^d^	−2.24 to − 1.59	< 10^−3^
	≥85 years	157,071	8.8	156,259	7.9	149,117	7.1	136,936	6.2	126,617	5.6	−2.49	−2.70 to − 2.28	< 10^−3^
	Women	454,173	12.2	429,707	11.3	396,996	10.4	349,987	9.3	314,041	8.3	−2.10	−2.43 to − 1.77	< 10^−3^
	Men	227,662	10.2	224,652	9.5	212,664	8.8	193,345	7.9	181,679	7.2	−1.93	−2.17 to − 1.68	< 10^−3^
**PIMs related to antihypertensive drugs**	Overall	426,370	7.4	424,198	7.0	407,924	6.6	400,491	6.4	377,277	6.0	−1.11	−1.33 to − 0.89	< 10^−3^
	75 to 84 years	283,332	7.1	271,646	6.6	252,385	6.2	239,416	6.0	220,602	5.4	−1.34^d^	−1.60 to − 1.07	< 10^−3^
	≥85 years	143,038	8.0	152,552	7.7	155,539	7.4	161,075	7.3	156,675	6.9	−0.77	−0.95 to − 0.60	< 10^−2^
	Women	280,767	7.9	277,127	7.5	266,357	7.1	260,549	6.9	244,282	6.4	−1.03^d^	−1.24 to −0.81	< 10^−3^
	Men	145,603	6.7	147,071	6.3	141,567	5.9	139,942	5.7	132,995	5.2	−1.26	−1.49 to − 1.03	< 10^−3^
**Use of 0 to 4 drugs**	Overall	1,415,619	24.6	1,498,596	24.7	1,509,145	24.5	1,578,364	25.5	1,665,214	26.3	0.36	0.02 to 0.70	0.06
	75 to 84 years	1,005,103	25.3	1,060,470	25.9	1,063,760	26.2	1,095,682	27.4	1,167,749	28.8	0.68^d^	0.40 to 0.96	< 10^−2^
	≥85 years	410,516	23.2	438,126	22.4	445,385	21.4	482,682	21.9	497,465	21.9	−0.29	−0.76 to 0.17	0.18
	Women	804,459	22.4	853,953	22.9	860,417	22.9	902,260	24.0	952,630	25.1	0.60^d^	0.26 to 0.94	0.02
	Men	611,160	27.9	644,643	27.4	648,728	27.0	676,104	27.6	712,584	28.1	0.05	−0.28 to 0.39	0.72
**Polypharmacy (5 to 9 drugs)**	Overall	2,603,266	44.9	2,825,898	46.5	2,911,467	47.3	2938,832	47.3	3,022,030	47.8	0.31	0.09 to 0.54	0.03
	75 to 84 years	1,830,378	45.7	1,927,386	46.9	1,930,113	47.4	1,894,051	47.3	1,918,708	47.3	0.17^d^	−0.03 to 0.37	0.11
	≥85 years	410,516	23.2	898,512	45.8	981,354	47.1	1,044,781	47.4	1,103,322	48.6	0.56	0.29 to 0.83	0.01
	Women	1,634,704	45.3	1,759,414	47.1	1,801,866	47.9	1,805,522	48.0	1,838,757	48.5	0.34	0.09 to 0.59	0.03
	Men	968,562	44.3	1,066,484	45.6	1,109,601	46.3	1,133,310	46.3	1,183,273	46.7	0.26	0.07 to 0.45	0.03
**Hyper-polypharmacy (≥ 10 drugs)**	Overall	1,758,760	30.5	1,744,248	28.8	1,731,249	28.2	1,689,777	27.2	1,640,911	25.9	−0.83	−1.00 to −0.66	< 10^−3^
	75 to 84 years	1,165,062	29.0	1,118,556	27.2	1,073,305	26.4	1,013,972	25.3	969,317	23.9	−0.99^d^	−1.17 to − 0.80	< 10^−3^
	≥85 years	593,698	33.3	625,692	31.9	657,944	31.5	675,805	30.7	671,594	29.6	−0.60	−0.78 to − 0.43	< 10^−2^
	Women	1,157,259	32.3	1,117,310	30.0	1,093,833	29.2	1,050,732	27.9	1,001,171	26.4	−1.03^d^	−1.24 to −0.82	< 10^−3^
	Men	601,501	27.9	626,938	27.0	637,416	26.7	639,045	26.1	639,740	25.2	−0.50	−0.63 to − 0.37	< 10^−2^
**≥ 3 CNS active drugs**	Overall	531,238	9.1	505,660	8.3	506,209	8.2	490,615	7.9	460,813	7.3	−1.07	−1.51 to −0.63	< 10^−2^
	75 to 84 years	349,484	8.6	322,955	7.8	311,817	7.6	292,112	7.3	268,465	6.6	−1.25	−1.69 to −0.81	< 10^−2^
	≥85 years	181,754	10.1	182,705	9.2	194,392	9.2	198,503	9.0	192,348	8.5	−0.81	−1.27 to − 0.35	0.02
	Women	412,114	11.5	389,357	10.5	387,558	10.3	373,699	9.9	348,670	9.2	−1.07	−1.51 to −0.64	< 10^−2^
	Men	119,124	5.5	116,303	5.0	118,651	5.0	116,916	4.8	112,143	4.4	−1.05	−1.51 to −0.59	< 10^−2^

^a^ Relative Annual Percent Change in a given year compared to the previous one

^b^ Sex-standardized

^c^ Age-standardized

^d ^95%CI for the difference in APC between groups did not include 0, indicating a difference between groups

PIMs related to BZDs were the most frequent PIMs over the study period (34.7% in 2011 and 26.9% in 2019). The prevalence of PIMs related to BZDs decreased by 1.34% per year (95%CI: [− 1.50 to − 1.17]), mainly because of a decrease in the dispensation of hypnotic Z-drugs and long-acting BZDs. Details about the prevalence and APC for each individual criterion are available in the online Supplementary Table 3.

The prevalence of PIMS related to ACH drugs decreased from 12.1 to 8.3% during the study period, with a relative decrease of 2.13% per year (95%CI: [− 2.47 to − 1.78]). First-generation antihistamines accounted for about three quarters of the PIMs related to ACH drugs.

Dispensation of PIMs related to NSAIDs decreased from 11.4 to 7.8% between 2011 and 2019, with a relative decrease of 2.04% per year (95%CI: [− 2.33 to − 1.74]) (Table 3). Repeated use of NSAIDs, defined as 3 reimbursements or more, was the main PIM related to NSAIDs (9.1% in 2011 and 6.0% in 2019).

PIMs related to AHT drugs had a prevalence of 7.4% in 2011 and 6.0% in 2019. The annual decrease in the prevalence of PIMs related to AHT drugs was estimated at 1.1% (95%CI: [− 1.33 to − 0.89]). The dispensation rate of central α-blockers and selective calcium channel blockers with immediate release were comparable (3.8 and 3.6% in 2011 respectively), although the dispensation of the former declined more rapidly over the study period, by 2.12% per year (95%CI: [− 2.51 to − 1.73]).

### Changes in polypharmacy and CNS-active drug prevalence

During the study period, the prevalence of hyper-polypharmacy (10 drugs or more) decreased from 30.5% in 2011 to 25.9% in 2019, whereas the prevalence of polypharmacy (5 to 9 drugs) increased from 44.9% in 2011 to 47.8% in 2019 (Table 3). Ultimately, the proportion of older persons not exposed to polypharmacy increased by 0.36% per year (95%CI: [0.02 to 0.70]), although this change was borderline significant (*p*-value for trend = 0.064). The prevalence of concomitant dispensation of three or more psychotropic drugs declined from 9.1% in 2011 to 7.3% in 2019 (Table 3).

### Differences according to age and sex

Although there was no major difference in the overall prevalence of PIMs according to age category in 2011, the steeper decrease in the 75–84 category over the study period resulted in a gap between age categories in 2019 (38.2% among the 75–84 and 42.1% among the 85+) (Table 3). During the study period, people aged 85+ were more likely to receive PIMs related to BZDs but less likely to receive PIMs related to NSAIDs or ACH drugs than people aged 75 to 84 years.

Although hyper-polypharmacy declined in both age categories, it still concerned 23.9% of the 75–84 category and 29.6% of the 85+ in 2019. People aged 85+ were more likely to receive at least three psychotropic drugs concomitantly than people aged 75 to 84 years, with a slower reduction over time.

There was no major difference between men and women in the direction of change in polypharmacy and PIM prevalence over the study period (except for tricyclic antidepressants and selective calcium blockers with immediate release). However, the magnitude of the change in prevalence differed between men and women, especially for the prevalence of hyper-polypharmacy which decreased twice as much in women as in men. Besides, women were constantly more exposed to both PIMs and polypharmacy than men (Table 3). It is of note that women were about 1.5 times more likely to receive BZDs than men over the study period (31.8% versus 19.6% in 2019).

## Discussion

This repeated cross-sectional analysis of comprehensive data on drug reimbursement in France shows that the prevalence of PIMs declined from 49 to 39% between 2011 and 2019 among people aged 75 years and older, corresponding to a relative decrease of 1.19% per year. Although the prevalence of PIMs declined in both sexes and both age categories, women and individuals aged 85 years and older were more likely to receive PIMs throughout the study period. BZDs were the most frequent PIMs, followed by ACH drugs, NSAIDs, and AHT drugs. All PIM categories followed a decreasing trend over the study period, especially ACH drugs and NSAIDs. It is of note that the decline in PIM prevalence over the study period went hand-in-hand with a slight decrease in hyper-polypharmacy.

The level of PIM prevalence in 2019 described in this study is in the upper range of published results on the prevalence of PIMs [10]. Methodological factors may contribute to the differences between studies. Notably, the use of claims data prevents drug underreporting issues, although self-medication is not captured. Additionally, the likelihood of observing a PIM is higher in studies such as ours, which consider medications dispensed over an entire year, compared with studies assessing medications taken on a single day or over a short period of time. Finally, our study population also included people living in nursing homes, who would be more likely to receive PIMs than old people living in community [26]. These methodological considerations aside, our result may nevertheless reflect the high level of exposure to PIMs in France and reminds us of the continuing need to reduce overuse and misuse of drugs. At the national level, our results are consistent with a previous work on a random sample of claims data, which reported that 53% of people aged 75 years or older had at least one PIM in 2007 [19].

The decreasing trend in PIM prevalence between 2011 and 2019 is consistent with the results of previous studies in western Europe and in the USA, although some differences between studies can be noted in the magnitude of reduction in PIMs [14, 16–18]. In France, a repeated cross-sectional study assessed the evolution of PIMs according to the 1997 Beers criteria between 1995 and 2004 in the east of France among a sample of volunteers, aged 65 years and older, from Centers for Preventive Medicine [18]. This study reported a relative decrease in PIM prevalence twice as high as our estimation, but the comparison with our study is limited by differences in study populations. Indeed, the study by Bongue et al. included individuals attending centers for preventive medicine, who may not be representative of the older population in France. Conversely, the relative decrease in PIM prevalence was twice as large in the present study as in a previous study in Germany which reported a decrease in PIMs from 26.4 to 23.1% between 2008 and 2016, also using the 2015 Beers criteria [14]. Finally, our results are consistent with those of a previous study in the USA reporting a 10.3% decrease over 4 years (from 45.5% in 2006 to 40.8% in 2010), whereas we observed a 20% decrease over 8 years [17].

Our study highlights the significant weight of BZDs in PIM prevalence in France, with levels of exposure higher than those reported in previous studies [16, 17]. At the European level, France ranked second in the use of BZDs in 2015 [27]. The decreasing trend in prescriptions of BZDs, especially of long acting BZDs, is thus encouraging, although it was less marked among women and people aged 85 years and over. The sex-difference in the prescription of BZDs may be related to the fact that women are more likely to be diagnosed with anxiety and mental disorders [28] and to seek health care than men [29]. Overall, the decreasing trend we observed may be related to the efforts made in France during the study period to reduce the prescription of BZDs. In particular, since 2011, general practitioners are encouraged to limit their prescriptions of BZDs through financial incentives from the national health insurance. Specific objectives regarding BZDs have been defined to limit new prescribing, to limit their duration (at 4 and 12 weeks for hypnotic Z-drugs and BZDs respectively), and to limit the prescription of long-acting BZDs in people aged 65 years and over. Moreover, restrictions were introduced in 2017 regarding the dispensing conditions of hypnotic Z-drugs, with the same precautions as for narcotics and the impossibility of using the same prescription twice.

Our results also show that PIMs related to NSAIDs and ACH drugs were reduced by about one third over the study period. This clear decrease may be due to a better awareness among prescribers of the risks of NSAIDs and ACH drugs in older people (notably renal failure for NSAIDs and falls for ACH drugs). In particular, specific tools have been developed in addition to PIM lists to assess the anticholinergic burden of medication among old people (anticholinergic risk scales) [30]. Additionally, there is no physical dependence on NSAIDs or ACH drugs contrary to BZDs, which may facilitate deprescribing or switching towards safer alternatives. In addition, the implementation of PIM lists in prescribing softwares and mobile applications, as well as the development of medication reconciliation in hospital, may help reducing dispensation of PIMs.

The analysis of the literature suggests a general trend of an increasing - or at best stable - prevalence of polypharmacy among older people [12, 13, 16, 31–33]. For instance, in Belgium, where the use of 5 or more drugs in 2015 among people aged 75 years and older was close to our results (49.5% in women and 50.9% in men), a relative increase of 89 and 80% was reported between 2000 and 2015, in men and women respectively [33]. Our study shows in contrast a decrease in the prevalence of hyper-polypharmacy. This result is accompanied by a slight increase in polypharmacy, which can be interpreted as a consequence of the decrease in hyper-polypharmacy. Indeed, people receiving 10 drugs or more may have been transferred to the polypharmacy category when their number of drugs decreased. This evolution towards a reduction of hyper-polypharmacy may have been fostered by the withdrawal of several drugs that were judged to be ineffective by the French National Health Authority (HAS) from the list of reimbursed drugs during the study period, for instance vasodilators containing ginkgo in 2012 and drugs for Alzheimer’s disease in 2018.

To our knowledge, this is the first study to measure the prevalence of PIMs and polypharmacy over time from comprehensive data in France. Strengths of this study include the assessment of PIMs according to internationally recognized lists of explicit criteria and the repeated measurements every two years between 2011 and 2019, which enabled us to investigate the dynamics of change over time. However, this study has some limitations related to the use of claims data, which cover only reimbursed drugs. This implies that, after a drug has been dispensed, there is no guarantee that it will be taken by the patient. Furthermore, some NSAIDs and ACH drugs are available over-the-counter, and could not be taken into account in the assessment of polypharmacy and PIMs. Changes in regulatory environment for medications during the study period occurred for dexchlorpheniramine, oxomemazine and ibuprofen 200 mg, although they only involved branded drugs and not generics for the latter two. However, given that reimbursed drugs represented 90% by value of the drug market in France in 2019 [34], we believe that the impact of self-medication on our results may be limited. Furthermore, the information available in claims data did not allow us to assess PIM criteria requiring complementary information such as indication or dosage, and we restricted our scope to PIM criteria that we could assess with as little uncertainty as possible. Other limitations include incomplete reporting of deaths in the DCIR, resulting in the inclusion of people deceased during the year (1.0 to 1.5% of the study population depending on the year considered), and missing information about the medication of residents in nursing homes with a pharmacy department (around 1200 out of 6990 nursing homes in France in 2017). Finally, we could not assess the effect of PIM reduction on the prevalence of assumed adverse outcomes related to PIMs (e.g. hospitalization related to medication problem).

## Conclusions

Dispensation of PIMs and hyper-polypharmacy have declined steadily in France since 2011, but levels remain high. Initial and continuous training of prescribers as well as the implementation of policies to regulate health care expenditure may have contributed to a modest improvement in prescribing practices for old people. However, the burden of BZDs remains a concern, especially in older women and in the oldest-old. As the population is growing old and the burden of multimorbidity is increasing, it is important to be able to monitor the evolution of the prevalence of PIMs and polypharmacy at the national level to evaluate public health policies and guide future decisions. Further work is needed to examine the levers of the reduction in potentially inappropriate prescriptions in order to enhance the decreasing trend of suboptimal prescribing for older people.

## Supplementary Information

**Additional file 1: Supplementary Table 1**. Selection of the study population for each study year.

**Additional file 2: Supplementary Table 2**. Sources, drugs and ATC codes, rationale and quality of the evidence according to the 2015 Beers criteria lists of the studied PIM criteria.

**Additional file 3: Supplementary Table 3**. Age- and sex-standardized prevalence of individual Potentially Inappropriate Medications (PIMs) dispensations by year and Annual Percent Change (APC), France, 2011–2019.

## Data Availability

In accordance with data protection legislation and the French regulation, the authors cannot publicly release the data from the French National Health Data System (*Système National des Données de Santé* – SNDS). However, any person or structure, public or private, for-profit or non-profit, is able to access SNDS data upon authorization from the French Data Protection Office (CNIL), in order to carry out a study, research or an evaluation in the public interest (https://www.snds.gouv.fr/SNDS/Processus-d-acces-aux-donnees and https://www.indsante.fr/). EPI-PHARE has permanent regulatory access to the data from the French National Health Data System (SNDS) via its constitutive bodies ANSM and CNAM. This permanent access is given in accordance with the French Decree No. 2016–1871 of December 26, 2016 relating to the processing of personal data called the “National Health Data System”, and French law articles Art. R. 1461–13 and 14. All requests in the database were made by duly authorized people. In accordance with the permanent regulatory access granted to EPI-PHARE via ANSM and CNAM, this work did not require the approval from the French Data Protection Authority (CNIL). The study was registered on the study register of EPI-PHARE concerning studies from SNDS data.

## Abbreviations

ACH: AntiCHolinergic
AHT: AntiHyperTensive
APC: Annual Percent Change
ATC: Anatomical Therapeutic Chemical
BZD: Benzodiazepines
CNS: Central Nervous System
DCIR: French health insurance claims data system (Données de Consommation Inter-Régime)
NSAIDs: Non-Steroidal Anti-Inflammatory Drugs
PIMs: Potentially Inappropriate Medications
STOPP: Screening Tool of Older People’s Prescriptions
