# Supplementary material for: Decreasing trends in potentially inappropriate medications in older people: a nationwide repeated cross-sectional study
Source: BMC Geriatr. 2021 Nov 2;21:621. doi: 10.1186/s12877-021-02568-1 (PMC8565059; doi:10.1186/s12877-021-02568-1)
Supplement: Supplementary file 1 — Additional file 1: Supplementary Table 1. Selection of the study population for each study year. [file 12877_2021_2568_MOESM1_ESM.docx]

**Supplementary Table 1. Selection of the study population for each study year.**

|  |  | **2 011** | **2 013** | **2 015** | **2 017** | **2 019** |
| --- | --- | --- | --- | --- | --- | --- |
| At least one medical care reimbursement during the year | N | 9,730,186 | 6,431,850 | 6,580,796 | 6,655,535 | 7,031,097 |
| Duplicates | N | 3,681,714 | 68,195 | 72,059 | 77,298 | 323,200 |
|  | Final | 6,048,472 | 6,363,655 | 6,508,737 | 6,578,237 | 6,707,897 |
| Undetermined gender | N | 694 | 567 | 520 | 545 | 484 |
|  | Final | 6,047,778 | 6,363,088 | 6,50,8217 | 6,577,692 | 6,707,413 |
| Abnormal date of birth | N | 24 | 8 | 6 | 3 | 3 |
|  | Final | 6,047,754 | 6,363,080 | 6,508,211 | 6,577,689 | 6,707,410 |
| Temporary identifiers | N | 3,051 | 0 | 13,685 | 21,071 | 29,943 |
|  | Final | 6,044,703 | 6,363,080 | 6,494,526 | 6,556,618 | 6,677,467 |
| Deceased during the year | N | 267,058 | 294,338 | 342,665 | 349,645 | 349,312 |
| Final population | N | 5,777,645 | 6,068,742 | 6,151,861 | 6,206,973 | 6,328,155 |
