# Supplementary material for: Decreasing trends in potentially inappropriate medications in older people: a nationwide repeated cross-sectional study
Source: BMC Geriatr. 2021 Nov 2;21:621. doi: 10.1186/s12877-021-02568-1 (PMC8565059; doi:10.1186/s12877-021-02568-1)
Supplement: Supplementary file 2 — Additional file 2: Supplementary Table 2. Sources, drugs and ATC codes, rationale and quality of the evidence according to the 2015 Beers criteria lists of the studied PIM criteria. [file 12877_2021_2568_MOESM2_ESM.docx]

Supplementary Table 2. Sources, drugs and ATC codes, rationale and quality of the evidence according to the 2015 Beers criteria lists of the studied PIM criteria.

| Indicator of potentially inappropriate medication | Source | Molecules | Anatomical Therapeutic Chemical (ATC) classification | | Rationale | Quality of evidence according to Beers’ criteria |
| --- | --- | --- | --- | --- | --- | --- |
| Anticholinergic drugs |  |  |  | | Increased risk of :  - Cognitive impairment, confusion, sedation  - Orthostatic hypotension, falls  - Urinary retention  - Dry mouth  - Constipation |  |
| Tricyclic antidepressants | Beers 2015 STOPP 2015 (D2) | Amoxapine  Amitryptyline  Clomipramine  Dosulepine  Doxepine  Imipramine  Maprotiline  Trimipramine | N06AF  N06AA | |  | High |
| Phenothiazines | Beers 2015  STOPP 2015 (D12) | Chlorpromazine  Clozapine  Cyamemazine  Fluphenazine  Propericiazine  Levomepromazine  Pipotiazine | N05AA  N05AB  N05AC | |  | Moderate |
| First-generation antihistamines | Beers 2015 | Alimemazine  Hydroxyzine  Bromphéniramine  Mequitazine  Oxomemazine | N05BB01  R06AB  R06AA  R06AD | |  | Moderate |
| Non-steroidal anti-inflammatory drugs (NSAIDs) |  |  |  | | Increased risk of gastrointestinal bleeding or peptic ulcer disease. Increased risk of renal failure. |  |
| Three or more dispensations of oral NSAIDs | Beers 2015  STOPP 2015 | Aceclofenac  Acide mefenamique  Acide niflumique  Acide tiaprofenique  Alminoprofene  Celecoxib  Dexketoprofene  Diclofenac  Etodolac  Etoricoxib  Fénoprofene  Flurbiprofene  Ibuprofene  Indometacine  Ketoprofene  Meloxicam  Nabumetone  Naproxene  Piroxicam  Sulindac  Ténoxicam | M01A  N02BA  Except for:  M01AX05 (glucosamine)  M01AX21 (diacereine)  M01AX25 (chondroitine sulfate)  M01AX26 (piascledine) | |  | Moderate |
| Concurrent use of two NSAIDs or more | STOPP 2015 (A3) |  |  | | No enhancement of efficacy | NA |
| NSAIDs in combination with vitamin K antagonist or non-vitamin k antagonist oral anti-coagulant | Beers 2015  STOPP 2015 (C10) | Warfarin  Coumarine  Fluindione  Acenocoumarol  Dabigatran  Rivaroxaban  Apixaban | B01AA  B01AE  B01AF | M01A  N02BA  Except for  M01AX05 (glucosamine)  M01AX21 (diacereine)  M01AX25 (chondroitine sulfate)  M01AX26 (piascledine) | Risk of gastro-intestinal bleeding | High |
| NSAIDs in combination with antiplatelet agents | STOPP 2015 (C11) | Clopidogrel  Acetylsalicylique acid  Ticlopidine  Dipyramidole  Prasugrel  Ticagrelor | B01AC | M01A  N02BA  Except for  M01AX05 (glucosamine)  M01AX21 (diacereine)  M01AX25 (chondroitine sulfate)  M01AX26 (piascledine) | Increased risk of peptic ulcer disease | NA |
| Any benzodiazepine (including hypnotic Z-drugs) | Beers 2015  STOPP 2015 (K1) |  | Long,short and intermediate acting benzodiazepines, hypnotic-Z drugs | | Increase sensitivity to benzodiazepines in old people.  For all benzodiazepines, increased risk of :   - - cognitive impairment - - delirium - - falls and fractures - - road accident |  |
| Other Benzodiazepines (Short- or intermediate- acting) | Beers 2015 |  | N05BA N05CD  (except long-acting benzodiazepines) | |  |  |
| Long-acting benzodiazepines  (half-life >=20h) | Beers 2015 | Bromazepam  Clobazam  Clonazepam  Potassium clorazepate  Diazepam  Estazolam  Ethyle loflazepate  Nordazepam  Prazepam  Nitrazepam | N03AE01  N05BA01  N05BA05  N05BA08  N05BA09  N05BA11  N05BA16  N05BA18  N05CD02  N05CD03  N05CD04  N05CD08 | | Decreased metabolism of long-acting benzodiazepines in old people, increased risk of adverse effects | Moderate |
| Hypnotic Z-drugs | Beers 2015  STOPP 2015 (K4) | Zolpidem  Zopiclone | N05CF | | Increased risk of hospitalization, minimal improvement in sleep latency and duration | moderate |
| Concurrent use of 2 or more benzodiazepines | Beers 2015  STOPP 2015 (A3) |  | Long,short and intermediate acting benzodiazepines | | No improvement of efficacy and increased risk of adverse effects | High |
| Concurrent use of benzodiazepines and hypnotic Z-drugs | Beers 2015 |  | Long,short and intermediate acting benzodiazepines + hypnotic Z-drugs | | Increased risk of adverse effects | High |
| Concurrent use of opioid receptor agonists and benzodiazepines | Beers 2015 |  | Long,short and intermediate acting benzodiazepines + N02A  N02BE71  N02AX02  N02AX52  N02AJ13 | | Increased risk of fall and overdose | High |
| Concurrent use of 3 central nervous system-active drugs or more | Beers 2015, 2019 | Long,short and intermediate acting benzodiazepines  Hypnotic-Zdrugs  Opioid receptor agonist  Tricyclic antidepressants  Serotonin-norepinephrine reuptake inhibitors  Selective serotonin reuptake inhibitors  Antipsychotics  Antiepileptics | Long,short and intermediate acting benzodiazepines; Hypnotic-Zdrugs; Opioid receptor agonist; Tricyclic antidepressants, Phenothiazine ;  N05A N05BE N05BC N05BX N05BD N05CA N05CB N05CC N05CX N05CE N06AX N06AG N06A N03 N06AB | | Increased risk of falls and fracture | Moderate |
| Antihypertensive drugs |  |  |  | |  |  |
| Central alpha-blockers | Beers 2015 | Clonidine  Méthyldopa  Moxonidine  rilménidine | C02AB  C02AC | | Higher risk of central nervous system effect:  - Orthostatic hypotension  - Bradycardia | Low |
| Selective calcium channel blockers with immediate release | Beers 2015 | Nifédipine  Nicardipine | C07FB03  C08CA05  C08CA04 | | Risk of hypotension and precipitating myocardial ischemia | High |
| Concurrent use of β-blockers and verapamil or diltiazem | STOPP 2015 (B3) | All β-bloquant drugs  Verapamil  Diltiazem | C07  C09BB10  C08DA01  C08DB01 | | Increased risk of heart block | NA |
