# Supplementary material for: Decreasing trends in potentially inappropriate medications in older people: a nationwide repeated cross-sectional study
Source: BMC Geriatr. 2021 Nov 2;21:621. doi: 10.1186/s12877-021-02568-1 (PMC8565059; doi:10.1186/s12877-021-02568-1)
Supplement: Supplementary file 3 — Additional file 3: Supplementary Table 3. Age- and sex-standardized prevalence of individual Potentially Inappropriate Medications (PIMs) dispensations by year and Annual Percent Change (APC), France, 2011–2019. [file 12877_2021_2568_MOESM3_ESM.docx]

**Supplementary Table 3. Age- and sex-standardized prevalence of individual Potentially Inappropriate Medications (PIMs) dispensations by year and Annual Percent Change (APC), France, 2011-2019.**

|  |  | **2011** | |  | |  | | **2013** | |  | |  | | **2015** | |  | |  | | **2017** | |  | |  | | **2019** | |  | |  | | **APC**§ | | **95%CI** | | **p-value for trend** | |  |
| --- | --- | --- | --- | --- | --- | --- | --- | --- | --- | --- | --- | --- | --- | --- | --- | --- | --- | --- | --- | --- | --- | --- | --- | --- | --- | --- | --- | --- | --- | --- | --- | --- | --- | --- | --- | --- | --- | --- |
|  |  | **(5,777,645)** | |  | |  | | **(6,068,742)** | |  | |  | | **(6,151,861)** | |  | |  | | **(6,206,973)** | |  | |  | | **(6,328,155)** | |  | |  | |  | |  | |  | |  |
|  |  | **No.** | | **%** | |  | | **No.** | | **%** | |  | | **No.** | | **%** | |  | | **No.** | | **%** | |  | | **No.** | | **%** | |  | |  | |  | |  | |  |
| **Short and intermediate benzodiazepines (≥3)** | Overall | 678,198 | | 11.8 | |  | | 763,023 | | 12.6 | |  | | 773,192 | | 12.6 | |  | | 793,171 | | 12.8 | |  | | 783,195 | | 12.4 | |  | | 0.24 | | [-0.35 to 0.84] | | 0.34 | |  |
|  | 75-84 | 424,450 | | 10.4 | |  | | 452,242 | | 10.9 | |  | | 433,86 | | 10.6 | |  | | 425,873 | | 10.6 | |  | | 407,717 | | 10.1 | |  | | -0.22* | | [-0.76 to 0.33] | | 0.36 | |  |
|  | ≥85 | 253,748 | | 14.1 | |  | | 310,781 | | 15.7 | |  | | 339,332 | | 16.2 | |  | | 367,298 | | 16.6 | |  | | 375,478 | | 16.5 | |  | | 0.82 | | [0.11 to 1.53] | | 0.05 | |  |
|  | Women | | 512,931 | | 14.5 | |  | | 568,919 | | 15.4 | |  | | 574,467 | | 15.4 | |  | | 586,078 | | 15.6 | |  | | 576,092 | | 15.2 | |  | | 0.24 | | [-0.30 to 0.78] | | 0.31 | |
|  | Men | | 165,267 | | 7.7 | |  | | 194,104 | | 8.4 | |  | | 198,725 | | 8.4 | |  | | 207,093 | | 8.5 | |  | | 207,103 | | 8.2 | |  | | 0.25 | | [-0.49 to 1.00] | | 0.41 | |
| **Long-acting benzodiazepines** | Overall | 889,845 | | 15.1 | |  | | 712,79 | | 11.6 | |  | | 648,913 | | 10.4 | |  | | 597,514 | | 9.6 | |  | | 551,783 | | 8.7 | |  | | -2.75 | | [-3.92 to -1.56] | | <10^-2^ | |  |
|  | 75-84 | 632,580 | | 15.6 | |  | | 496,95 | | 12.0 | |  | | 444,78 | | 10.9 | |  | | 403,82 | | 10.1 | |  | | 372,742 | | 9.2 | |  | | -2.63 | | [-3.84 to -1.41] | | <10^-2^ | |  |
|  | ≥85 | 257,265 | | 14.3 | |  | | 215,84 | | 10.9 | |  | | 204,133 | | 9.7 | |  | | 193,694 | | 8.8 | |  | | 179,041 | | 7.9 | |  | | -3.03 | | [-4.15 to -1.90] | | <10^-2^ | |  |
|  | Women | 653,124 | | 17.9 | |  | | 522,072 | | 13.9 | |  | | 473,868 | | 12.5 | |  | | 433,517 | | 11.5 | |  | | 397,885 | | 10.5 | |  | | -2.69 | | [-3.81 to -1.55] | | <10^-2^ | |  |
|  | Men | 236,721 | | 10.8 | |  | | 190,718 | | 8.1 | |  | | 175,045 | | 7.3 | |  | | 163,997 | | 6.7 | |  | | 153,898 | | 6.1 | |  | | -2.90 | | [-4.20 to -1.58] | | <10^-2^ | |  |
| **Hypnotic Z-drugs** | Overall | 896,676 | | 15,5 | |  | | 891,835 | | 14.7 | |  | | 842,538 | | 13.7 | |  | | 758,231 | | 12.2 | |  | | 649,504 | | 10.3 | |  | | -2.18 | | [-3.00 to -1.35] | | <10^-2^ | |  |
|  | 75-84 | 594,943 | | 14,7 | |  | | 572,512 | | 13.9 | |  | | 525,162 | | 12.9 | |  | | 458,666 | | 11.4 | |  | | 385,96 | | 9.5 | |  | | -2.29 | | [-3.13 to -1.44] | | <10^-2^ | |  |
|  | ≥85 | 301,733 | | 16,9 | |  | | 319,323 | | 16.2 | |  | | 317,376 | | 15.2 | |  | | 299,565 | | 13.6 | |  | | 263,544 | | 11.6 | |  | | -2.00 | | [-2.80 to -1.19] | | <10^-2^ | |  |
|  | Women | 621,540 | | 17,4 | |  | | 611,231 | | 16.4 | |  | | 575,635 | | 15.4 | |  | | 516,469 | | 13.7 | |  | | 440,012 | | 11.6 | |  | | -2.11 | | [-2.91 to -1.31] | | <10^-2^ | |  |
|  | Men | 275,136 | | 12,8 | |  | | 280,604 | | 12.1 | |  | | 266,903 | | 11.2 | |  | | 241,762 | | 9.9 | |  | | 209,492 | | 8.3 | |  | | -2.30 | | [-3.16 to -1.43] | | <10^-2^ | |  |
| **Concurrent use of at least two benzodiazepines** | Overall | 130,943 | | 2.2 | |  | | 110,888 | | 1.8 | |  | | 104,338 | | 1.7 | |  | | 104,253 | | 1.7 | |  | | 93,571 | | 1.5 | |  | | -1.89 | | [-2.98 to -0.79] | | 0.02 | |  |
|  | 75-84 | 91,120 | | 2.2 | |  | | 74,524 | | 1.8 | |  | | 67,851 | | 1.7 | |  | | 66,396 | | 1.7 | |  | | 58,643 | | 1.4 | |  | | -2.03 | | [-3.21 to -0.83] | | 0.02 | |  |
|  | ≥85 | 39,823 | | 2.2 | |  | | 36,364 | | 1.8 | |  | | 36,487 | | 1.7 | |  | | 37,857 | | 1.7 | |  | | 34,928 | | 1.5 | |  | | -1.70 | | [-2.65 to -0.75] | | 0.02 | |  |
|  | Women | 102,824 | | 2.8 | |  | | 85,771 | | 2.3 | |  | | 80,309 | | 2.1 | |  | | 79,72 | | 2.1 | |  | | 71,14 | | 1.9 | |  | | -1.93 | | [-3.06 to -0.79] | | 0.02 | |  |
|  | Men | 28,119 | | 1.3 | |  | | 25,117 | | 1.1 | |  | | 24,029 | | 1.0 | |  | | 24,533 | | 1.0 | |  | | 22,431 | | 0.9 | |  | | -1.78 | | [-2.75 to -0.80] | | 0.02 | |  |
| **Concurrent use of benzodiazepines and hypnotic Z-drugs** | Overall | 264,035 | | 4.5 | |  | | 260,132 | | 4.3 | |  | | 245,115 | | 4.0 | |  | | 220,631 | | 3.5 | |  | | 188,429 | | 3.0 | |  | | -2.18 | | [-2.98 to -1.37] | | <10^-2^ | |  |
|  | 75-84 | 177,846 | | 4.4 | |  | | 167,539 | | 4.0 | |  | | 151,787 | | 3.7 | |  | | 131,906 | | 3.3 | |  | | 110,02 | | 2.7 | |  | | -2.48 | | [-3.25 to -1.71] | | <10^-2^ | |  |
|  | ≥85 | 86,189 | | 4.8 | |  | | 92,593 | | 4.7 | |  | | 93,328 | | 4.4 | |  | | 88,725 | | 4.0 | |  | | 78,409 | | 3.5 | |  | | -1.74 | | [-2.63 to -0.85] | | 0.01 | |  |
|  | Women | 199,840 | | 5.5 | |  | | 194,686 | | 5.2 | |  | | 182,787 | | 4.9 | |  | | 164,149 | | 4.4 | |  | | 139,162 | | 3.7 | |  | | -2.16 | | [-2.95 to -1.36] | | <10^-2^ | |  |
|  | Men | 64,195 | | 3.0 | |  | | 65,446 | | 2.8 | |  | | 62,328 | | 2.6 | |  | | 56,482 | | 2.3 | |  | | 49,267 | | 1.9 | |  | | -2.25 | | [-3.10 to -1.39] | | <10^-2^ | |  |
| **Concurrent use of opioid receptor agonists and benzodiazepines** | Overall | 403,664 | | 6.8 | |  | | 356,646 | | 5.8 | |  | | 350,871 | | 5.7 | |  | | 344,151 | | 5.5 | |  | | 311,502 | | 4.9 | |  | | -1.53 | | [-2.36 to -0.70] | | 0.02 | |  |
|  | 75-84 | 274,880 | | 6.7 | |  | | 233,062 | | 5.6 | |  | | 221,235 | | 5.4 | |  | | 210,542 | | 5.2 | |  | | 186,81 | | 4.6 | |  | | -1.77 | | [-2.67 to -0.85] | | 0.01 | |  |
|  | ≥85 | 128,784 | | 7.1 | |  | | 123,584 | | 6.2 | |  | | 129,636 | | 6.2 | |  | | 133,609 | | 6.0 | |  | | 124,692 | | 5.5 | |  | | -1.19 | | [-1.91 to -0.47] | | 0.02 | |  |
|  | Women | 318,210 | | 8.8 | |  | | 279,706 | | 7.5 | |  | | 272,904 | | 7.3 | |  | | 265,389 | | 7.1 | |  | | 237,956 | | 6.3 | |  | | -1.58 | | [-2.38 to -0.78] | | 0.01 | |  |
|  | Men | 85,454 | | 3.9 | |  | | 76,94 | | 3.3 | |  | | 77,967 | | 3.3 | |  | | 78,762 | | 3.2 | |  | | 73,546 | | 2.9 | |  | | -1.36 | | [-2.30 to -0.41] | | 0.03 | |  |
| **First-generation antihistamine** | Overall | 543,023 | | 9.3 | |  | | 523,074 | | 8.6 | |  | | 457,605 | | 7.4 | |  | | 395,569 | | 6.4 | |  | | 351,714 | | 5.6 | |  | | -2.84 | | [-3.24 to -2.43] | | <10^-3^ | |  |
|  | 75-84 | 382,759 | | 9.5 | |  | | 363,514 | | 8.8 | |  | | 311,374 | | 7.6 | |  | | 266,904 | | 6.7 | |  | | 238,67 | | 5.9 | |  | | -2.65* | | [-3.04 to -2.26] | | <10^-3^ | |  |
|  | ≥85 | 160,264 | | 9.0 | |  | | 159,56 | | 8.1 | |  | | 146,231 | | 7.0 | |  | | 128,665 | | 5.8 | |  | | 113,044 | | 5.0 | |  | | -3.23 | | [-3.71 to -2.74] | | <10^-3^ | |  |
|  | Women | 367,244 | | 10.1 | |  | | 348,157 | | 9.3 | |  | | 304,154 | | 8.1 | |  | | 261,549 | | 7.0 | |  | | 229,489 | | 6.1 | |  | | -2.82 | | [-3.20 to -2.44] | | <10^-3^ | |  |
|  | Men | 175,779 | | 8.1 | |  | | 174,917 | | 7.5 | |  | | 153,451 | | 6.4 | |  | | 134,02 | | 5.5 | |  | | 122,225 | | 4.8 | |  | | -2.86 | | [-3.35 to -2.38] | | <10^-3^ | |  |
| **Tricyclic antidepressants** | Overall | 147,557 | | 2.5 | |  | | 157,884 | | 2.5 | |  | | 156,053 | | 2.5 | |  | | 153,189 | | 2.5 | |  | | 150,693 | | 2.4 | |  | | -0.24 | | [-0.64 to 0.15] | | 0.18 | |  |
|  | 75-84 | 107,063 | | 2.6 | |  | | 112,474 | | 2.7 | |  | | 108,509 | | 2.6 | |  | | 103,848 | | 2.6 | |  | | 100,782 | | 2.5 | |  | | -0.32 | | [-0.77 to 0.12] | | 0.14 | |  |
|  | ≥85 | 40,494 | | 2.2 | |  | | 45,41 | | 2.3 | |  | | 47,544 | | 2.3 | |  | | 49,341 | | 2.2 | |  | | 49,911 | | 2.2 | |  | | -0.15 | | [-0.39 to 0.10] | | 0.20 | |  |
|  | Women | 115,294 | | 3.1 | |  | | 121,256 | | 3.2 | |  | | 119,029 | | 3.1 | |  | | 116,034 | | 3.1 | |  | | 112,856 | | 3.0 | |  | | -0.33 | | [-0.68 to 0.03] | | 0.09 | |  |
|  | Men | 32,263 | | 1.5 | |  | | 36,628 | | 1.6 | |  | | 37,024 | | 1.5 | |  | | 37,155 | | 1.5 | |  | | 37,837 | | 1.5 | |  | | 0.02 | | [-0.51 to 0.55] | | 0.94 | |  |
| **Phenothiazine** | Overall | 57,308 | | 1.0 | |  | | 58,539 | | 1.0 | |  | | 53,425 | | 0.9 | |  | | 48,524 | | 0.8 | |  | | 44,015 | | 0.7 | |  | | -1.98 | | [-2.51 to -1.45] | | <10^-2^ | |  |
|  | 75-84 | 37,593 | | 0.9 | |  | | 37,555 | | 0.9 | |  | | 33,28 | | 0.8 | |  | | 29,47 | | 0.7 | |  | | 26,494 | | 0.7 | |  | | -1.98 | | [-2.58 to -1.38] | | <10^-2^ | |  |
|  | ≥85 | 19,715 | | 1.1 | |  | | 20,984 | | 1.1 | |  | | 20,145 | | 1.0 | |  | | 19,054 | | 0.9 | |  | | 17,521 | | 0.8 | |  | | -1.99 | | [-2.51 to -1.46] | | <10^-2^ | |  |
|  | Women | 39,661 | | 1.1 | |  | | 39,988 | | 1.1 | |  | | 36,347 | | 1.0 | |  | | 32,652 | | 0.9 | |  | | 29,556 | | 0.8 | |  | | -2.01 | | [-2.54 to -1.48] | | <10^-2^ | |  |
|  | Men | 17,647 | | 0.8 | |  | | 18,551 | | 0.8 | |  | | 17,078 | | 0.7 | |  | | 15,872 | | 0.6 | |  | | 14,459 | | 0.6 | |  | | -2.00 | | [-2.63 to -1.37] | | <10^-2^ | |  |
| **Oral NSAIDs† (≥3)** | Overall | 547,777 | | 9.1 | |  | | 516,71 | | 8.3 | |  | | 474,649 | | 7.6 | |  | | 417,678 | | 6.7 | |  | | 377,57 | | 6.0 | |  | | -2.29 | | [-2.54 to -2.04] | | <10^-3^ | |  |
|  | 75-84 | 426,560 | | 10.6 | |  | | 398,689 | | 9.6 | |  | | 363,733 | | 8.9 | |  | | 317,341 | | 7.9 | |  | | 285,013 | | 7.0 | |  | | -2.18* | | [-2.47 to -1.88] | | <10^-3^ | |  |
|  | ≥85 | 121,217 | | 6.8 | |  | | 118,021 | | 6.0 | |  | | 110,916 | | 5.3 | |  | | 100,337 | | 4.5 | |  | | 92,557 | | 4.1 | |  | | -2.78 | | [-2.98 to -2.58] | | <10^-3^ | |  |
|  | Women | 374,066 | | 10.0 | |  | | 348,753 | | 9.1 | |  | | 318,648 | | 8.4 | |  | | 278,144 | | 7.4 | |  | | 248,699 | | 6.6 | |  | | -2.29 | | [-2.56 to -2.01] | | <10^-3^ | |  |
|  | Men | 173,711 | | 7.8 | |  | | 167,957 | | 7.1 | |  | | 156,001 | | 6.5 | |  | | 139,534 | | 5.7 | |  | | 128,871 | | 5.1 | |  | | -2.31 | | [-2.52 to -2.09] | | <10^-3^ | |  |
| **Concurrent use of 2 or more NSAIDs** | Overall | 38,545 | | 0.6 | |  | | 34,795 | | 0.6 | |  | | 31,175 | | 0.5 | |  | | 26,327 | | 0.4 | |  | | 23,624 | | 0.4 | |  | | -2.92 | | [-3.16 to -2.67] | | <10^-3^ | |  |
|  | 75-84 | 30,984 | | 0.8 | |  | | 27,9 | | 0.7 | |  | | 24,854 | | 0.6 | |  | | 20,951 | | 0.5 | |  | | 18,734 | | 0.5 | |  | | -2.72* | | [-2.94 to -2.49] | | <10^-3^ | |  |
|  | ≥85 | 7,561 | | 0.4 | |  | | 6,895 | | 0.3 | |  | | 6,321 | | 0.3 | |  | | 5,376 | | 0.2 | |  | | 4,89 | | 0.2 | |  | | -3.65 | | [-4.08 to -3.23] | | <10^-3^ | |  |
|  | Women | 26,499 | | 0.7 | |  | | 23,881 | | 0.6 | |  | | 21,177 | | 0.6 | |  | | 17,676 | | 0.5 | |  | | 15,643 | | 0.4 | |  | | -2.90 | | [-3.17 to -2.62] | | <10^-3^ | |  |
|  | Men | 12,046 | | 0.5 | |  | | 10,914 | | 0.5 | |  | | 9,998 | | 0.4 | |  | | 8,651 | | 0.4 | |  | | 7,981 | | 0.3 | |  | | -2.84 | | [-3.13 to -2.55] | | <10^-3^ | |  |
| **NSAIDs in combination with antiplatelet agents** | Overall | 223,139 | | 3.8 | |  | | 219,112 | | 3.6 | |  | | 207,522 | | 3.4 | |  | | 183,331 | | 2.9 | |  | | 160,722 | | 2.5 | |  | | -2.15 | | [-2.84 to -1.44] | | <10^-2^ | |  |
|  | 75-84 | 164,813 | | 4.1 | |  | | 159,954 | | 3.9 | |  | | 150,252 | | 3.7 | |  | | 131,463 | | 3.3 | |  | | 115,728 | | 2.9 | |  | | -1.96* | | [-2.65 to -1.26] | | <10^-2^ | |  |
|  | ≥85 | 58,326 | | 3.3 | |  | | 59,158 | | 3.0 | |  | | 57,27 | | 2.7 | |  | | 51,868 | | 2.4 | |  | | 44,994 | | 2.0 | |  | | -2.69 | | [-3.39 to -2.00] | | <10^-2^ | |  |
|  | Women | 134,936 | | 3.7 | |  | | 130,654 | | 3.5 | |  | | 122,428 | | 3.2 | |  | | 106,239 | | 2.8 | |  | | 90,818 | | 2.4 | |  | | -2.29 | | [-3.08 to -1.50] | | <10^-2^ | |  |
|  | Men | 88,203 | | 4.0 | |  | | 88,458 | | 3.7 | |  | | 85,094 | | 3.5 | |  | | 77,092 | | 3.1 | |  | | 69,904 | | 2.8 | |  | | -1.95 | | [-2.53 to -1.37] | | <10^-2^ | |  |
| **NSAIDs in combination with vitamin K antagonist or non-vitamin k antagonist oral anti-coagulant** | Overall | 26,290 | | 0.5 | |  | | 30,07 | | 0.5 | |  | | 30,49 | | 0.5 | |  | | 33,323 | | 0.5 | |  | | 37,722 | | 0.6 | |  | | 1.42 | | [0.80 to 2.05] | | <10^-2^ | |  |
|  | 75-84 | 19,202 | | 0.5 | |  | | 21,231 | | 0.5 | |  | | 21,267 | | 0.5 | |  | | 22,748 | | 0.6 | |  | | 25,774 | | 0.6 | |  | | 1.39 | | [0.79 to 1.99] | | <10^-2^ | |  |
|  | ≥85 | 7,088 | | 0.4 | |  | | 8,839 | | 0.5 | |  | | 9,223 | | 0.4 | |  | | 10,575 | | 0.5 | |  | | 11,948 | | 0.5 | |  | | 1.30 | | [0.61 to 2.00] | | 0.01 | |  |
|  | Women | 14,373 | | 0.4 | |  | | 16,353 | | 0.4 | |  | | 16,456 | | 0.4 | |  | | 18,113 | | 0.5 | |  | | 20,132 | | 0.5 | |  | | 1.55 | | [0.97 to 2.14] | | <10^-2^ | |  |
|  | Men | 11,917 | | 0.5 | |  | | 13,717 | | 0.6 | |  | | 14,034 | | 0.6 | |  | | 15,21 | | 0.6 | |  | | 17,59 | | 0.7 | |  | | 1.22 | | [0.60 to 1.85] | | 0.01 | |  |
| **Central alpha-** **blockers** | Overall | 223,538 | | 3.8 | |  | | 212,334 | | 3.5 | |  | | 192,759 | | 3.1 | |  | | 183,568 | | 2.9 | |  | | 161,25 | | 2.6 | |  | | -2.12 | | [-2.51 to -1.73] | | <10^-3^ | |  |
|  | 75-84 | 151,790 | | 3.8 | |  | | 139,979 | | 3.4 | |  | | 123,607 | | 3.0 | |  | | 114,157 | | 2.8 | |  | | 98,873 | | 2.4 | |  | | -2.25 | | [-2.66 to -1.83] | | <10^-3^ | |  |
|  | ≥85 | 71,748 | | 4.0 | |  | | 72,355 | | 3.7 | |  | | 69,152 | | 3.3 | |  | | 69,411 | | 3.1 | |  | | 62,377 | | 2.7 | |  | | -1.96 | | [-2.34 to -1.57] | | <10^-3^ | |  |
|  | Women | 152,550 | | 4.2 | |  | | 142,406 | | 3.8 | |  | | 127,87 | | 3.4 | |  | | 119,931 | | 3.2 | |  | | 103,14 | | 2.7 | |  | | -2.29* | | [-2.71 to -1.88] | | <10^-3^ | |  |
|  | Men | 70,988 | | 3.2 | |  | | 69,928 | | 3.0 | |  | | 64,889 | | 2.7 | |  | | 63,637 | | 2.6 | |  | | 58,11 | | 2.3 | |  | | -1.80 | | [-2.17 to -1.43] | | <10^-3^ | |  |
| **Selective calcium channel blockers with immediate release** | Overall | 204,818 | | 3.6 | |  | | 214,927 | | 3.6 | |  | | 219,237 | | 3.6 | |  | | 223,559 | | 3.6 | |  | | 222,458 | | 3.5 | |  | | -0.07 | | [-0.22 to 0.09] | | 0.33 | |  |
|  | 75-84 | 131,820 | | 3.3 | |  | | 132,71 | | 3.2 | |  | | 130,448 | | 3.2 | |  | | 128,421 | | 3.2 | |  | | 124,866 | | 3.1 | |  | | -0.31* | | [-0.53 to -0.10] | | 0.03 | |  |
|  | ≥85 | 72,998 | | 4.1 | |  | | 82,217 | | 4.2 | |  | | 88,789 | | 4.2 | |  | | 95,138 | | 4.3 | |  | | 97,592 | | 4.3 | |  | | 0.28 | | [0.12 to 0.43] | | 0.02 | |  |
|  | Women | 130,536 | | 3.7 | |  | | 137,608 | | 3.7 | |  | | 141,989 | | 3.8 | |  | | 145,575 | | 3.9 | |  | | 145,884 | | 3.8 | |  | | 0.25* | | [0.09 to 0.41] | | 0.02 | |  |
|  | Men | 74,282 | | 3.4 | |  | | 77,319 | | 3.3 | |  | | 77,248 | | 3.2 | |  | | 77,984 | | 3.2 | |  | | 76,574 | | 3.0 | |  | | -0.64 | | [-0.83 to -0.45] | | <10^-2^ | |  |
| **Concurrent use of Beta-bloquant and verapamil or diltiazem** | Overall | 21,165 | | 0.4 | |  | | 19,434 | | 0.3 | |  | | 17,244 | | 0.3 | |  | | 15,007 | | 0.2 | |  | | 13,24 | | 0.2 | |  | | -2.94 | | [-3.06 to -2.82] | | <10^-3^ | |  |
|  | 75-84 | 14,997 | | 0.4 | |  | | 13,232 | | 0.3 | |  | | 11,418 | | 0.3 | |  | | 9,625 | | 0.2 | |  | | 8,305 | | 0.2 | |  | | -3.22* | | [-3.34 to -3.09] | | <10^-3^ | |  |
|  | ≥85 | 6,168 | | 0.3 | |  | | 6,202 | | 0.3 | |  | | 5,826 | | 0.3 | |  | | 5,382 | | 0.2 | |  | | 4,935 | | 0.2 | |  | | -2.55 | | [-2.77 to -2.32] | | <10^-3^ | |  |
|  | Women | 13,104 | | 0.4 | |  | | 12,04 | | 0.3 | |  | | 10,666 | | 0.3 | |  | | 9,345 | | 0.2 | |  | | 8,154 | | 0.2 | |  | | -2.79* | | [-2.97 to -2.60] | | <10^-3^ | |  |
|  | Men | 8,061 | | 0.4 | |  | | 7,394 | | 0.3 | |  | | 6,578 | | 0.3 | |  | | 5,662 | | 0.2 | |  | | 5,086 | | 0.2 | |  | | -3.22 | | [-3.36 to -3.08] | | <10^-3^ | |  |

§ Relative Annual Percent Change in a given year compared to the previous one

† Non-steroidal anti-inflammatory drugs

* 95%CI for the difference in APC between men and women or between 75-84 years and ≥85 years did not include 0 meaning there is a difference between the APC
